# Effects of Composite Supplement Containing Collagen Peptide and Ornithine on Skin Conditions and Plasma IGF-1 Levels—A Randomized, Double-Blind, Placebo-Controlled Trial

**DOI:** 10.3390/md16120482

**Published:** 2018-12-03

**Authors:** Naoki Ito, Shinobu Seki, Fumitaka Ueda

**Affiliations:** Pharmaceutical and Healthcare Research Laboratories, Research and Development Management Headquarters, FUJIFILM Corporation, 577, Ushijima, Kaisei-machi, Ashigarakami-gun, Kanagawa 258-8577, Japan; shinobu.seki@fujifilm.com (S.S.); fumitaka.ueda@fujifilm.com (F.U.)

**Keywords:** collagen peptide, ornithine, skin elasticity, transepidermal water loss, growth hormone, insulin-like growth factor-1

## Abstract

Aging-associated changes of skin conditions are a major concern for maintaining quality of life. Therefore, the improvement of skin conditions by dietary supplementation is a topic of public interest. In this study, we hypothesized that a composite supplement containing fish derived-collagen peptide and ornithine (CPO) could improve skin conditions by increasing plasma growth hormone and/or insulin-like growth factor-1 (IGF-1) levels. Twenty-two healthy Japanese participants were enrolled in an 8-week double-blind placebo-controlled pilot study. They were assigned to either a CPO group, who were supplemented with a drink containing CPO, or an identical placebo group. We examined skin conditions including elasticity and transepidermal water loss (TEWL), as well as plasma growth hormone and IGF-1 levels. Skin elasticity and TEWL were significantly improved in the CPO group compared with the placebo group. Furthermore, only the CPO group showed increased plasma IGF-1 levels after 8 weeks of supplementation compared with the baseline. Our results might suggest the novel possibility for the use of CPO to improve skin conditions by increasing plasma IGF-1 levels.

## 1. Introduction

The major role of skin is the protection of our body from external stimuli. Skin also plays an important role in maintaining homeostasis, including protection against loss of moisture and adjustment of body temperature [1]. Furthermore, aging-associated changes of skin conditions, such as the increased wrinkles and decreased skin elasticity, are a major concern for maintaining quality of life. Skin is composed of epidermis, dermis, and subcutaneous tissue. Collagen and elastin in the dermis maintain the structure of skin and create its elasticity [2,3]. Notably, the fibrous protein collagen, which plays a major role in maintaining the mechanical strength of skin, constitutes the majority of the dermis. The collagen molecule is formed by the three polypeptides, named alpha chains. The alpha chains are composed of high levels of glycine, hydroxyproline, proline and alanine [4]. Thus, collagen has unique amino acid composition. These collagen molecules assemble to form collagen fibrils by cross linking. Then, collagen fibrils assemble to form large collagen fiber. There are several types of collagen, and type I and type III collagen are the most abundant collagen in the skin [5]. Because collagen synthesis decreases with aging, decreased collagen content is one of the major causes of aging-associated changes of skin conditions [6,7]. As skin aging and nutrition states are linked [8], the improvement of skin conditions by dietary supplementation is a topic of increasing public interest [9].

The effects of growth hormone, a peptide hormone secreted from the pituitary gland (especially during the first few hours of sleep), cover a broad range of biological phenomena including cell growth, proliferation, regeneration, and metabolism [10,11,12]. For skin, severe growth hormone deficiency results in early aging, such as wrinkling and dryness [13]. In addition to the direct effects of growth hormone to several tissues, it also elicits indirect effects mediated by insulin-like growth factor-1 (IGF-1) [12]. Plasma IGF-1 levels correlate with plasma growth hormone levels, as production of IGF-1 by the liver is stimulated by growth hormone [14,15]. Similar to growth hormone, IGF-1 activates cell growth in several tissues including bone [16], muscle [17], and skin [18,19], whereby it contributes to both epidermal or dermal skin development and maintenance. Secretion of growth hormone is decreased with aging, suggesting that aging-associated changes of skin conditions are mediated, at least in part, by decreased levels of growth hormone, or its associated decrease of IGF-1 [20]. Thus, these previous studies suggest that aging-associated changes of skin conditions might be improved by increasing growth hormone and/or IGF-1 levels.

In recent years, many products containing collagen or a denatured form of collagen have been used for a wide variety of purposes, including cosmetics and food. With regard to food or supplements, the effects of collagen have been controversial, as orally ingested native collagen or its partially hydrolyzed form, gelatin, are not efficiently absorbed [21]. However, several lines of evidence revealed the beneficial role of collagen-derived small peptides, which exhibit high absorbability compared with native collagen [21,22], for a wide variety of tissues including bone [23], joint [24], muscle [25], tendon [26], and skin [27,28,29] in humans. Collagen has been isolated from many marine, brackish water and freshwater sources such as fishes [30,31,32] and mollusks [33,34,35]. Compared with collagen peptide derived from land animals, collagen peptide derived from these aquatic sources has unique molecular and biological properties for amino acid composition, antioxidant activity, neuroprotective activity and anti-skin aging activity, because of low temperature and/or high salt condition in the surrounding environment [36,37,38,39]. Furthermore, a previous study showed that collagen derived from sea- and freshwater-rainbow trout had quite similar amino acid composition and molecular weight properties [40]. Collagen derived from two marine demosponges, *Axinella cannabina* and *Suberites carnosus*, collected from the Aegean and the Ionian Seas, respectively, had low imino acid content, and showed lower or similar denaturation temperatures compared with collagen derived from other marine organisms such as tropical fish [41], suggesting the universal properties of collagen derived from aquatic sources. In addition to collagen peptide, ornithine is a non-essential, non-protein amino acid contained in various foods such as freshwater clams. Recent studies have highlighted ornithine as a functional food for improving sleep quality [42] and recovery from fatigue [43,44] in human.

The beneficial effects of collagen peptide for skin conditions have been analyzed by several groups both in rodents [39] and humans [27,28,29,45]. However, to our knowledge, the effects of collagen peptide, ornithine or the combined effects of collagen peptide and ornithine (CPO) on the increase of growth hormone and/or IGF-1 levels, and the subsequent improvements of skin conditions have not been investigated. A previous study showed the increase of plasma growth hormone levels after ingestion of ornithine [46], though its relationship to the improvements of skin conditions have not been investigated. In this study, we anticipated the combinational effects of fish-derived collagen peptide and ornithine on skin conditions, and plasma growth hormone and/or IGF-1 levels in healthy Japanese people. We hypothesized that orally administered CPO induced an increase of plasma growth hormone and/or IGF-1 levels, which exerted subsequent improvements of skin conditions including elasticity, moisture and transepidermal water loss (TEWL).

## 2. Results

### 2.1. Participants

**Figure 1:**
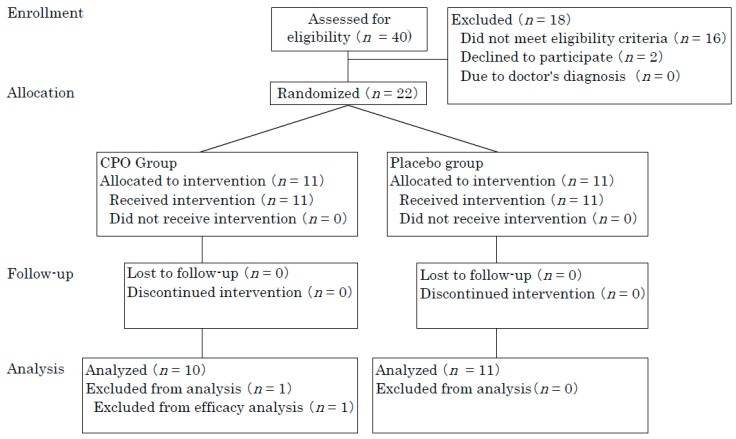
Flow diagram of participants.

Forty participants were recruited from the Osaka area, of whom 22 participants (aged from 31 to 48 years, 18 females and 4 males) exhibiting low skin moisture and elasticity were enrolled. Participants were recruited from September to October 2017. Included participants were assigned to either the CPO group (*n* = 11) or placebo group (*n* = 11). All participants completed the study. One participant in the CPO group was excluded from analysis because of aberrant blood ureic acid levels both before and after supplementation (Figure 1).

Finally, 21 participants (aged from 31 to 48 years, 17 females and 4 males) were analyzed. Thus, per protocol set analysis was performed. Statistically significant differences between originally included participants and finally analyzed participants were not observed for baseline scores including age, skin elasticity, and moisture. This study consisted of an 8-week administration period from October to December 2017. CPO and placebo groups were matched according to age, gender, body mass index (BMI), skin elasticity, and moisture at baseline (Table 1 and Table 2).

Average ingestion rate was 100 ± 0% and 99.8 ± 0.5% in CPO and placebo groups, respectively. All subjects had a more than 98% ingestion rate. No statistically significant differences were observed for ingestion rate between CPO group and placebo groups.

### 2.2. Skin Conditions

**Figure 2:**
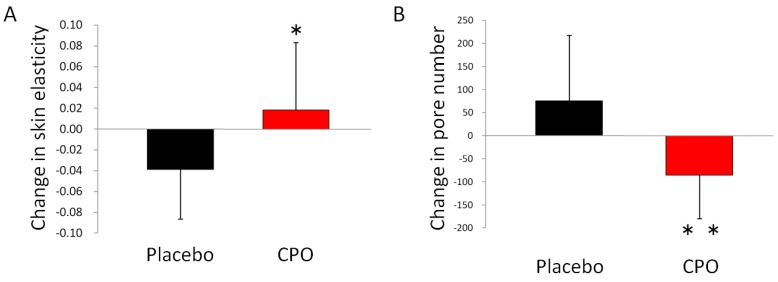
Dietary supplementation with collagen peptide and ornithine increased skin elasticity, and decreased the number of pores. Change in skin elasticity from baseline (**A**), and change in the number of pores from baseline (**B**). * *p* < 0.05 and ** *p* < 0.01 by unpaired *t*-test. Error bars indicate SD.

The aim of this study was to evaluate the effects of CPO on skin conditions including elasticity, moisture, and TEWL. These skin conditions were measured at baseline and after 8 weeks of supplementation (Table 2). TEWL was significantly attenuated in the CPO group compared with the placebo group after 8 weeks of supplementation. For skin elasticity and VISIA analysis, no statistically significant differences were observed between raw values for CPO and placebo groups. However, placebo group showed a decrease of elasticity, from 0.818 ± 0.05 at baseline to 0.779 ± 0.06 after 8 weeks of supplementation. On the other hand, CPO groups showed an increase of elasticity, from 0.766 ± 0.07 at baseline to 0.784 ± 0.07 after 8 weeks of supplementation (Table 2). By analyzing the changes from the baseline, we observed statistically significant differences between the placebo and CPO groups (−0.039 ± 0.047 in placebo group, and 0.018 ± 0.065, Figure 2A). In addition to elasticity, a significantly reduced change in the number of skin pores was also observed after 8 weeks of supplementation in the CPO group compared with the placebo group (Figure 2B).

In addition, only the placebo group showed significantly decreased collagen scores compared with baseline using the DermaLab test (placebo group, from 45.9 ± 17.9 to 40.9 ± 14.6, *p* = 0.027; CPO group, from 41.1 ± 15.2 to 38.9 ± 13.4, *p* = 0.097). No statistically significant differences in moisture or superficial pH were observed between CPO and placebo groups.

### 2.3. Plasma Growth Hormone and Insulin-Like Growth Factor-1 (IGF-1) Levels

**Figure 3:**
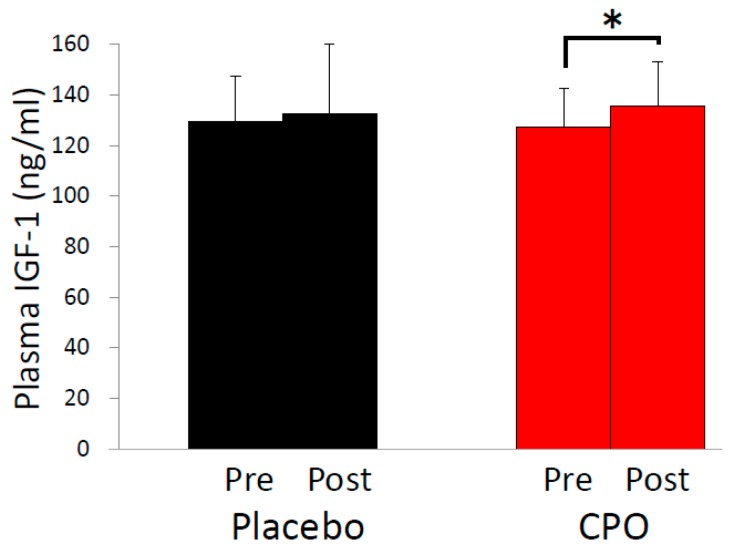
Dietary supplementation with collagen peptide and ornithine increased plasma insulin-like growth factor-1 (IGF-1) levels. Comparison of plasma IGF-1 levels before and after 8-week supplementation with collagen peptide and ornithine (CPO) (right) or with placebo (left). * *p* < 0.05 by paired *t*-test. Error bars indicate SD.

As aging-associated changes of skin condition are partially mediated by decreased levels of growth hormone [13], we analyzed plasma levels of growth hormone before and 30, 60, 120, 150, 180 and 240 min after supplementation. Growth hormone levels before supplementation were more than two-fold higher in the CPO group compared with the placebo group (0.65 ± 0.49 ng/mL in the placebo group vs. 1.41 ± 1.70 ng/mL in the CPO group, *p* = 0.778), suggesting that comparison of growth hormone levels between CPO and placebo groups was unworthy of evaluation. Indeed, we observed no significant differences of plasma growth hormone levels in the CPO group compared with the placebo group. We also evaluated IGF-1 levels at baseline and after 8 weeks of supplementation because IGF-1 levels reflect growth hormone secretion [15]. No statistically significant difference between CPO and placebo groups was observed. However, a statistically significant increase of IGF-1 levels from the baseline was observed only in the CPO group (Figure 3), suggesting that the improvements of skin conditions by CPO were mediated by the increase of IGF-1 levels, at least in part.

### 2.4. Clinical Safety

We observed neither adverse events nor severe changes in scores for general biochemical examination of blood or hematologic tests. Adverse events related to the ingestion of CPO were not observed. Thus, safety concerns were not observed.

## 3. Discussion

To our knowledge, our study is the first report to demonstrate the combined effects of CPO on skin conditions, plasma growth hormone and IGF-1 levels. We found that dietary supplementation of CPO improved skin elasticity and TEWL. TEWL was increased in the placebo group, while the CPO group showed no increase, indicating that the seasonal increase of TEWL was prevented by CPO (Table 2). As TEWL is linked to the barrier function of skin [1], this result suggests a protective effect of CPO for skin barrier function. In addition to TEWL, we observed a statistically significant difference between placebo and CPO groups in the changes of elasticity from baseline. Elasticity in the placebo group was decreased, while the CPO group showed increased elasticity, suggesting that the seasonal decrease in elasticity was prevented by CPO (Figure 2A). Furthermore, we observed increased IGF-1 levels only in the CPO group, suggesting that the improvements of skin conditions were mediated, at least in part, by increased IGF-1 levels (Figure 3).

The effects of collagen peptide on the improvements of skin moisture, elasticity, wrinkles, ultraviolet-induced erythema and ultraviolet-induced pigmented spots were previously revealed by several groups [28,29,45,47,48,49]. Furthermore, it has been previously reported that oral administration of marine collagen peptide derived from the skin of Nile Tilapia enhanced the process of wound healing [32]. Marine collagen peptide derived from Chum Salmon also promoted cutaneous wound healing [50,51]. Our study reinforced the beneficial effects of fish-derived collagen peptide to maintain or improve skin conditions such as elasticity and TEWL. Furthermore, we performed combined administration of collagen peptide and ornithine. Similar to collagen peptide, a previous study showed that ornithine enhanced wound healing effects by upregulating collagen synthesis in mice [52], suggesting that both collagen peptide and ornithine contribute to the improvements of skin conditions. In light of the independent effects of collagen peptide and ornithine, we hypothesized that skin conditions would be improved by the synergistic effects of CPO to increase growth hormone and/or IGF-1 levels, as described in the Introduction. Indeed, we observed the increased IGF-1 levels only in the CPO group. Between-group differences for TEWL and elasticity reinforced our hypothesis. However, because the effects of ornithine on skin conditions have not been investigated in humans, we could not conclude that the improvements of skin conditions observed in this study were derived from either the sole effects of collagen peptide or ornithine, or the synergistic effects of CPO. Furthermore, because we did not evaluate the sole effects of collagen peptide in this study, we could not conclude that the previously observed improvements of skin conditions including moisture and TEWL by collagen peptide [28,29,45,47,48,49] was enhanced by co-administration of ornithine. A comparison of the sole effects of ornithine, collagen peptide, and CPO is required to evaluate the synergist effects of CPO in the future.

As described in the Materials and Methods section, we employed a cutometer with a 6-mm diameter probe, suggesting that the improvement of skin elasticity reflected the state of relatively deep skin areas, such as the dermis. Generally, improvement of skin barrier function leads to the attenuation of TEWL, which results in subsequent improvement of the dermis environment [53,54]. In addition to this general understanding, increased IGF-1 levels in the CPO group suggested that the attenuation of TEWL occurred through the improvement of the dermal environment, which can result in the activation of dermal fibroblasts. Increased collagen scores in the CPO group, as measured by the DermaLab test, support the notion of CPO improving the dermal environment. Furthermore, previous studies have shown that the increased IGF-1 levels or treatment with collagen peptides leads to the activation of dermal fibroblasts [55,56]. Activated dermal fibroblasts construct the firm structure of the basement membrane, which is required for stable adherence of epidermal cells to the basement membrane. This stable adherence maintains an adequate balance between proliferation and differentiation of epidermal cells, leading to the enhancement of barrier function and subsequent attenuation of TEWL [57]. However, further study is required to determine how CPO improved skin elasticity and TEWL, as well as the relationship between improvements of elasticity and TEWL. Furthermore, we focused on the improvement of skin elasticity exclusively in the neck because only a thin muscle is present under neck skin [58]. However, as the effects of IGF-1 would not be restricted only to neck skin, we suspect that the positive effects observed in this study would be applicable to other areas of skin.

In this study, we hypothesized that CPO improved skin condition by increasing the secretion of growth hormone and/or IGF-1. Indeed, we observed increased plasma IGF-1 levels, which reflect increased secretion of growth hormone in the CPO group [15]. However, we did not observe an apparent increase of growth hormone levels immediately after CPO supplementation. One possibility for this result is that CPO enhanced secretion of growth hormone levels during the night, as we required participants to take CPO before bed time and growth hormone is secreted during non-rapid eye movement sleep. Thus, analysis for the effect of CPO on growth hormone secretion during sleep merits future investigation to potentially explain increased IGF-1 levels elicited by CPO supplementation.

Our study has several limitations. Even though the sample size was limited, which rated this study as a pilot trial, we observed improvement of skin elasticity, TEWL and increase of IGF-1 levels by CPO, suggesting the strong effects of CPO. Ornithine is found in freshwater clams, a traditional food for Japanese people. Furthermore, fish dishes are favored by Japanese people. We prohibited participants from continuously ingesting a functional food with identical or similar effects as the active ingredient of the test food. However, we did not estimate the exact dietary intake of CPO by participants; thus, the effects of CPO were potentially underestimated or overestimated. We hypothesized that combined supplementation of CPO elicited increased growth hormone and/or IGF-1 levels, which was followed by the improvement of the skin condition. In fact, we observed improvements of both elasticity and TEWL. However, the only intra-group difference was observed for increased plasma IGF-1 levels in the CPO group. Thus, precise mechanisms underlying how CPO improved the condition of skin, the specific contributions of CPO, and potential synergistic effects of CPO were not elucidated. Further analysis or a large-scale study is required to examine how CPO influences skin conditions.

## 4. Materials and Methods

### 4.1. Study Design, Randomization and Blinding

This study was a randomized, double-blind, placebo-controlled, parallel-group comparison trial to evaluate the effects of CPO dietary supplementation on skin conditions, plasma growth hormone and IGF-1 levels in healthy Japanese participants. Equal numbers of participants were allocated to active and placebo groups. This study, which was approved by the Kenshokai Ethical Review Board (Approved Number: 20170927-1) and registered in the UMIN Clinical Trials Registry (ID: UMIN000028924), followed the Declaration of Helsinki and Ethical Guidelines for Medical and Health Research Involving Human Subjects. Participants, clinicians, and practitioners were blinded. Practitioners performed intervention, outcome measurements, and analysis. Clinicians performed safety evaluation. According to our previous independent trial to evaluate skin TEWL or minimum erythema dose by dietary supplementation of astaxanthin in 10 healthy people [9,59], we set the required sample size as 10. The evaluation of skin elasticity was the primary outcome, while secondary outcome measures included other skin conditions such as skin moisture and TEWL, as well as plasma levels of growth hormone and IGF-1, and safety evaluation. Participants were enrolled and randomly allocated into the CPO or placebo group using a random number table with consideration of sex, age, skin elasticity, and moisture by practitioners. Allocation was concealed until all participants completed the tests.

### 4.2. Participants

Participants aged from 30 to less than 50 years in the Osaka area were included in this study. Every participant received an explanation of the objectives and details of this study, and provided written informed consent themselves. This study consisted of an 8-week ingestion period from October to December 2017. Participants meeting the following criteria were included in the study: (1) aged from 30 to 49 years at the time informed consent was provided; (2) exhibited relatively low levels of blood IGF-1; (3) exhibited relatively low skin moisture and skin elasticity; (4) BMI was less than 25; (5) capable of visiting the administrative facility on every inspection day; and (6) provided written informed consent for involvement in this trial themselves. Participants with the following criteria were excluded from the study: (1) continuous ingestion of a functional food or supplement; (2) continuous ingestion of a functional food or quasi-medicine with identical or similar effects as the active ingredient in test food; (3) frequent ingestion of food which rich in the same active ingredient in test food or ingestion of these kinds of food during the 3 days before and after trial initiation, or during the last 3 days of the trial; (4) worked a night shift or day and night shifts; (5) receiving medical treatment or prophylactic treatment, or diagnosed with the need for medical treatment; (6) presence of skin disease or abnormality in skin condition, such as atopic dermatitis; (7) exhibited apparent change of skin condition that was not related to the intake of test food at the end of the trial compared with the initiation; (8) history of severe disease or abnormality in glucose metabolism, lipid metabolism, liver function, kidney function, or cardiovascular system function including heart, respiratory tract, endocrine system and nerve system function, or psychiatric disorder; (9) exhibiting anemia, or felt sick as a result of blood collection; (10) history of alcoholism or drug addiction; (11) risk of food allergy; (12) exhibited apparent abnormality in blood test or were positive for hepatitis B antigen or hepatitis C virus antibody in trial duration, including the screening period; (13) pregnant or lactating when informed consent was provided, or hoped to become pregnant during the trial; (14) involvement in another trial within 4 weeks prior to this trial, or participation in another concurrent trial; and (15) otherwise judged to be inappropriate for this trial by the clinician responsible for this trial.

### 4.3. Supplement Formulation

One 30-mL CPO drink contained 10 g of fish-derived collagen peptide (FUJIFILM, Tokyo, Japan), 400 mg of ornithine, and other ingredients including vitamin C, acidifier, and sweetener. To prepare fish-derived collagen peptide, gelatin was extracted from the fish scales, almost all from Tilapia, by hot water extraction. Gelatin was then digested by food-processing protease to prepare fish-derived collagen peptide. Total amount of hydroxyproline in fish-derived collagen peptide, which was analyzed by the hydrolysis of collagen peptide and subsequent high performance liquid chromatography, was 1100 mg in one CPO drink. The weight-average molecular weight of the collagen peptide was 2000 to 3000 Da. This drink contained less than 9 mg of potassium and 0.3 mg of magnesium. The placebo drink contained the same amount of vitamin C, acidifier, and sweetener, but did not contain CPO. One bottle was administered every day before bedtime for 8 weeks. On the test initiation day, the test drink was administered in the daytime because of the measurement of growth hormone levels. CPO and placebo drinks, including the bottle, were indistinguishable by shape, taste, or color. The dosage of collagen peptide was determined according to the previous report that showed the dose-dependent increase of blood hydroxyproline content by the intake of 2, 10 and 25 g of collagen peptide in humans [60]. In addition, the dose and the duration of this study were based on our previous study focusing on the effects of collagen peptide (UMIN: 000016587). The dosage of ornithine was based on an earlier study focusing on the effects of ornithine on sleep quality [42].

### 4.4. Evaluation of Skin Condition

For evaluation of skin conditions, we measured skin elasticity using a Cutometer MPA580 with a 6-mm diameter probe (Courage and Khazaka Electronic GmbH, Cologne, Germany). A 6-mm diameter probe was used, as opposed to a 2- or 4-mm diameter probe, to analyze functional changes in relatively deep areas of skin, such as the dermis. Furthermore, we analyzed skin elasticity at the neck because only thin muscles, such as the sternocleidomastoid muscle, are under the skin, making the neck a suitable area to analyze skin elasticity compared with the cheek, which mount on facial muscle [58]. To evaluate skin moisture, TEWL, and superficial pH at the cheek, a Corneometer (Courage and Khazaka Electronic GmbH, Cologne, Germany), VAPO SCAN AS-VT100RS (ASCH JAPAN Co., Tokyo, Japan), and Skin-pH-Meter PH905 (Courage & Khazaka Electronic GmbH, Cologne, Germany) were used, respectively. Skin conditions were also evaluated by VISIA Evolution (Canfield Scientific Ltd., Parsippany, NJ, USA), which analyzed spots, wrinkles, pores, texture, porphyrins, UV spots, red areas and brown spots from the picture of the participant’s face [61], and DermaLab (Cortex Technology, Hadsund, Denmark), which analyzed echo-graphic density of subcutaneous tissue including epidermis, dermis and subcutaneous fat and calculated collagen score from its ultrasound image. All measurements of skin condition were evaluated in a testing room with stable temperature (21 ± 1 °C) and humidity (50 ± 5%).

### 4.5. Blood Sampling and Safety Evaluation

Serum was obtained at baseline and after 8 weeks of supplementation, and blood IGF-1 levels were evaluated. To analyze the increases of plasma growth hormone levels, serum was obtained at 0, 60, 120, 150, 180, and 240 min after ingestion of test food. Plasma IGF-1 and growth hormone levels were measured by LSI Medience Co, Ltd. (Osaka, Japan) General biochemical examination of blood and hematologic tests were performed for safety evaluation.

### 4.6. Statistical Analysis

All results were presented as mean ± standard deviation (SD). Normality was analyzed by the Shapiro–Wilk test. If the data showed a normal distribution, differences between CPO and placebo groups were assessed by an unpaired *t*-test, and intra-group changes were evaluated by paired *t*-test. If the data did not show a normal distribution, we performed a Wilcoxon signed-rank test to analyze inter-group differences, and Wilcoxon rank sum test to analyze between-group differences. No additional analysis was performed. Probabilities less than 5% (* *p* < 0.05 and ** *p* < 0.01) were considered to be statistically significant. Statistical analyses were performed with JMP (version 13).

## Figures and Tables

**Table 1:** Baseline characteristics of participants who completed 8-week test.

	Placebo (*n* = 11)	Collagen Peptide and Ornithine (CPO) (*n* = 10)	*p* Value
Age (mean ± standard deviation (SD))	40.4 ± 5.2	40.0 ± 6.8	0.89
Female, *n* (%)	9 (81.8)	8 (80.0)	
Body mass index (BMI) (mean ± SD)	20.6 ± 1.8	21.2 ± 2.2	0.55

**Table 2:** Score of skin conditions.

		Week	
		0	8
Skin Conditions	Group	Mean ± SD	Mean ± SD
Moisture (A.U.)	Placebo	57.3 ± 13.3	60.9 ± 10.7
	CPO	58.5 ± 7.4	60.3 ± 8.2
TEWL (g/m^2^ × h)	Placebo	14.4 ± 4.3	16.2 ± 3.4
	CPO	11.3 ± 4.1	11.5 ± 4.2 *
Elasticity	Placebo	0.818 ± 0.05	0.779 ± 0.06
	CPO	0.766 ± 0.07	0.784 ± 0.07
Skin-pH	Placebo	5.96 ± 0.23	6.00 ± 0.24
	CPO	6.03 ± 0.24	6.15 ± 0.23
DermaLab Collagen Score	Placebo	45.9 ± 17.9	40.9 ± 14.6
	CPO	41.1 ± 15.2	38.9 ± 13.4
VISIA Spots	Placebo	99.9 ± 38.1	96.1 ± 37.6
	CPO	83.3 ± 39.0	75.1 ± 34.6
VISIA Wrinkles	Placebo	172.3 ± 100.5	186.9 ± 124.5
	CPO	186.2 ± 108.2	209.5 ± 106.9
VISIA pores	Placebo	670.2 ± 328.8	745.8 ± 354.2
	CPO	848.4 ± 404.6	763.5 ± 452.7
VISIA Texture	Placebo	1972.0 ± 1171.7	1755.8 ± 1172.9
	CPO	1773.4 ± 1276.9	1610.5 ± 1243.7
VISIA Porphyrins	Placebo	719.9 ± 709.6	607.6 ± 585.6
	CPO	694.4 ± 402.4	868.2 ± 648.8
VISIA Spots	Placebo	193.3 ± 63.7	202.0 ± 56.9
	CPO	199.7 ± 56.4	209.9 ± 43.1
Red Areas	Placebo	49.5 ± 14.6	49.3 ± 14.8
	CPO	42.9 ± 17.2	36.4 ± 17.3
Brown Spots	Placebo	123.5 ± 58.5	125.3 ± 61.1
	CPO	104.1 ± 47.4	109.6 ± 50.5

* *p* < 0.05 vs. placebo.
